# Purchase of medications without prescription in Peru: a cross-sectional population-based study

**DOI:** 10.12688/f1000research.15886.2

**Published:** 2019-02-22

**Authors:** Akram Hernández-Vásquez, Christoper A. Alarcon-Ruiz, Deysi Díaz-Seijas, Luisa Magallanes-Quevedo, Diego Rosselli

**Affiliations:** 1 Universidad San Ignacio de Loyola, Centro de Excelencia en Estudios Económicos y Sociales en Salud, Lima, 15024, Peru; 2 Universidad San Ignacio de Loyola, Unidad de Investigación para la Generación y Síntesis de Evidencias en Salud, Lima, 15024, Peru; 3 Universidad Nacional Mayor de San Marcos, Lima, 15081, Peru; 4 Department of Clinical Epidemiology and Biostatistics, Pontificia Universidad Javeriana, Bogota, 110311, Colombia

**Keywords:** Nonprescription Drugs, Self Medication, Drug Utilization, Drug Seeking Behavior

## Abstract

**Background: **Low availability of medicines in health services, self-medication, inadequate use of medicines, and inadequate dispensing practices in pharmacies are frequent problems in Peru. We aimed to evaluate how frequent the purchase of medications without medical prescription is in Peru, and which factors are associated with this practice.

**Methods: **We conducted a secondary analysis of the 2016 ENSUSALUD national survey data. Purchase of one or more medicines that require a prescription was measured as a dichotomous variable. Crude and adjusted prevalence ratios (PR) and their 95% confidence intervals (95% CI) were calculated using Poisson regressions model with robust variance to assess the association of purchasing of medicines that require prescriptions with sociodemographic factors.

**Results: **There were 3858 participants in the dataset. The prevalence of purchasing medications without prescriptions was 47.2%. History of having previously consumed the same medication (31.6%), and the delay in receiving an appointment at health facilities (26.9%) were the main reasons to buy medications without a prescription. Regarding the recommendation of the medication purchased, the advice of the pharmacy, and remembering a previous old prescription, were the most frequent reasons (38.3%, and 25.9%, respectively). On the multivariable analysis, users that buy medications without prescription were more likely to be of aged 25-44; reside in the Jungle  and Highlands regions; and self-consumption of the purchase. Individuals with *Seguro Integral de Salud* (Comprehensive Health Insurance) were less likely to buy medications without prescription.

**Conclusions: **There is a high prevalence of prescription requiring medication being bought without one from pharmacies in Peru. It is necessary to include the evaluation of consumer patterns to develop strategies with the aim to regulate the consumption of prescription drugs in the Peruvian population.

## Introduction

Self-medication is the use of medicines to treat symptoms or disorders identified by the users without prescription, or the intermittent or continued use of a medicine prescribed by a physician to treat recurring or chronic symptoms or illnesses of the user or their family members, especially children or elderly people ^1^. It is a common practice both in developed and developing countries that may cause potential risks due to an incorrect self-diagnosis, failures in treatment, risk of overdose, medical interactions, severe adverse effects, among others ^2, 3^.

Several studies show that patterns of self-medication widely vary among populations, and these patterns are influenced by multiple factors, such as age, sex, income, expenses, orientation to self-care, level of education, medical knowledge, satisfaction with health services, and perception of illness ^1, 4^. A systematic review of publications from different countries show a prevalence of self-medication ranging between 12.7% and 71% ^5^.

In Peru, important processes of health reform have been developed, meaning advances to achieve an increased coverage of universal insurance in health, and defense of users’ rights ^6^. However, in 2014, it was reported that 30.6% of patients had an insufficient access to medicines which patients requested in pharmacies of Peruvian health facilities. Furthermore, 20.7% of users of pharmacies of public health facilities where they had been treated were told to purchase medicines in an external pharmacy ^7^. Other studies have shown that 13% of adults who purchased antibiotics for children under five years bought them in private pharmacies without medical prescription. These results could demonstrate a low availability of medicines in health facilities, which added to self-medication, an inadequate use of medicines, and inadequate dispensing practices in pharmacies, represent a critical problem in the Peruvian health system with prevalence of self-medication ranging from 10.5% to 87.8%, during 2000 and 2015 ^8– 11^.

In Peru, the National Survey on User Satisfaction of Health Services ( *Encuesta Nacional de Satisfacción de Usuarios en Salud*, ENSUSALUD) has been conducted since 2014, this survey includes information related to the purchase of medicines and its results are nationally representative ^12^; however, to our best knowledge, studies using this survey have not been conducted. Such studies might assess a very frequent practice in the country such as the purchase of medicines without medical prescription.

To that effect, the objective of this study was to assess the prevalence of purchasing medicines which required a medical prescription in Peru without prescription, to know their characteristics, and to identify factors related to this practice.

## Methods

### Study design and data sources

We conducted a secondary analysis of database from the Module of Pharmacies’ Users of the ENSUSALUD 2016 prepared by the National Superintendency of Health ( *Superintendencia Nacional de Salud*, SUSALUD) and the National Institute of Statistics and Data Processing ( *Instituto Nacional de Estadística e Informática*, INEI) ^12^. All complete cases of people older than 15 years were included for the analysis.

ENSUSALUD includes questionnaires (available from the SUSALUD website) addressed to health professionals, executives of health services, and users of pharmacies and health services, and their objective is to monitor and assess the functioning and performance of the Peruvian health system, by studying the main participants in health care processes provided by health facilities ^13^. The survey is conducted yearly using face-to-face questionnaires and in its 2016 edition, this survey took place from May 13 to July 9. ENSUSALUD is nationally representative, by applying a probabilistic, stratified and two-stages sampling with a simple random selection in the first stage and a systematic selection in the second stage, and using a level of confidence of 95% and a sampling error of +/-5%. ENSUSALUD obtains information of people older than 15 years who went to buy some medicine for themselves, their partners or their son/daughter in a pharmacy located up to two blocks around the health facilities selected to participate in the study.

The databases of ENSUSALUD 2016 public and can be obtained SUSALUD website.

### Variables

For this analysis all the questions were obtained from the ENSUSALUD questionnaire. The dependent variable “purchase of prescription medicines without medical prescription” was determined by using the question “Did you buy this/these medicine(s) using a prescription?” for each one of the medicines purchased in order to determine if the interviewee had purchased at least one prescription medicine without medical prescription. The classification of each one of the medicines dependent on if they required a medical prescription or not for their sale was conducted by two investigators independently, based on Health Registration of Pharmaceutical Products of the General Bureau for Medicines, Drugs, and Inputs of Peru, in which official information of the national regulatory agency is included for all authorized medicines sold in Peru. The independent variables included are: sex, age group, level of education, type of health insurance, geographical area of residency, language, consumption of purchased products, and request of prescription by a pharmacist.

With regards to independent variables, it is important to point out that in Peru health insurance can correspond to one of the four subsystems: public system, which subsidizes health services to low-income population; social security system (EsSalud), which provides formal workers and their dependants with services; health system of armed forces and police (FF.AA.); and private system, available to people who can pay directly for a service or by a private insurance company ^14, 15^. The country is divided into 24 political-administrative departments which are distributed in three natural regions: Coast region, adjacent to the Pacific Ocean (included Metropolitan Lima, capital city of the country); Highlands region, where the Andes are located; and Jungle region, which is part of the Amazon rainforest.

Additionally, the following questions were included in the analysis: “Why did you buy medicines without prescription?” and “Who recommended you these medicines?” with the purpose of knowing the reasons for purchasing the medicine(s) and people responsible for recommending medicines purchased by survey respondents.

### Statistical analysis

The analysis of data was conducted using Stata® software v14.2 (Stata Corporation, College Station, Texas, USA). In the first stage, characteristics of study populations were described by absolute frequencies and percentages, their weighted proportions were also estimated with a 95% confidence interval based on the purchase of medicines without prescription. The second stage was composed by a bivariate and multivariate statistical analysis, for that reason generalized linear models (GLM) of *Poisson* family and the *log* linking function were used to determine the association between the purchase of medicine without prescription and the independent variables. For the multivariate analysis, independent variables with a minimal association (p<0.2) with the dependent variable were included, and reasons of prevalence with 95% confidence interval (95% CI) were reported. Variables included in multivariate analysis were sex, age group, level of education, type of health insurance, geographical area of residency, language, consumption of purchased products, and request of prescription by a pharmacist. The characteristics of sampling of the survey were specified, they include weightings according to strata, expansion factor, and design; for this purpose, the *svy* command was used. For all statistical testing a p value of <0.05 was considered statistically significant.

### Ethical considerations

This study did not require any approval of an ethics committee, because it is a secondary analysis of a database of free access and public domain which does not identify survey respondents or pharmacies where purchases were made.

## Results

The Module of Pharmacies’ Users of ENSUSALUD included a total of 3858 survey respondents (expanded population: 3,529,791), of that 1892 participants purchased at least one prescription medicine without medical prescription, which represents a prevalence of 47.2% (95% CI: 45.3-49.1). The average age of the survey respondents who purchased without medical prescription was 38.4 ± 14.1. The survey respondents with private health insurance (57%), with university education (51.4%), who are residents of the Highlands region (57.1%) purchased more often without a medical prescription ( Table 1). Additionally, the pharmacist in 69.6% of the purchases did not enquire for a prescription.

**Table 1.** General characteristics of the population included in the study and the proportion of users who purchased prescription medicines without medical prescription in Peru, *Encuesta Nacional de Satisfacción de Usuarios en Salud* (ENSUSALUD) 2016 (N=3858).

Characteristic	Absolute number and weighted proportion of survey respondents *	Weighted proportion of users who purchased at least one medicine which require a prescription for its sale * (n=1892)	
	n (%)	%	(95% CI)
**Total**	3858 (100)	47.2	(45.3-49.1)
**Sex**			
Male	1698 (43.9)	50.5	(47.6-53.3)
Female	2160 (56.1)	44.7	(42.2-47.2)
**Age (years)**			
Average (SD)	39.4+-14.2		38.4+-14.1
**Age group**			
15–24	502 (13.2)	51.9	(46.6-57.2)
25–44	2151 (53.1)	49.2	(46.7-51.8)
45–59	810 (22.1)	41.9	(37.9-46.1)
60 and more	395 (11.6)	42.9	(37.2-48.7)
**Level of education**			
Until elementary school	494 (12.2)	45.0	(39.9-50.2)
High school	1508 (38.5)	45.2	(42.2-48.1)
Non-university higher education	787 (19.9)	46.4	(42.2-50.6)
University education	1069 (29.5)	51.4	(47.8-55.0)
**Type of health insurance**			
Without insurance	1198 (33.1)	53.0	(49.6-56.3)
Comprehensive Health Insurance (SIS)	1333 (32.6)	40.8	(37.7-44.0)
EsSalud	1182 (29.4)	47.2	(43.7-50.7)
Private and other	89 (3.1)	57.0	(45.0-68.2)
FF.AA.	56 (1.7)	41.4	(27.6-56.6)
**Geographical area of** residency **			
Metropolitan Lima	517 (29.5)	36.9	(32.9-41.2)
Other areas of Coast region	1130 (22.3)	40.4	(37.0-43.9)
Highlands region	780 (14.5)	57.1	(53.0-61.1)
Jungle region	1431 (33.8)	56.5	(53.5-59.4)
**Language**			
Spanish	3779 (98.6)	47.1	(45.1-49.0)
Other	79 (1.4)	59.1	(46.3-70.8)
**Consumption of** **purchased products**			
For other people	1208 (30.6)	35.2	(32.1-38.5)
For themselves	2650 (69.4)	52.5	(50.2-54.8)
**Request of prescription** **by a pharmacist**			
No	2780 (69.6)	64.7	(62.5-66.9)
Yes	1078 (30.4)	7.2	(5.7-9.0)

*Weighted mass and specifications of sampling of ENSUSALUD 2016 were included **In Peru, there are 24 political-administrative departments which are distributed in three natural regions: Coast region, adjacent to the Pacific Ocean (included Metropolitan Lima, capital city of the country); Highlands region, where Andes are located; and Jungle region, which is part of the Amazon rainforest. SD: Standard deviation. FF.AA: Health System of Armed Forces and Police.

The precedent of previously using the medicine (31.6%), the delay in obtaining an appointment for health care (26.9%), and the delay for health care in health centers (21.5%) were the main causes of purchasing prescription medicines without medical prescription. Regarding the individual who recommended the purchased medicine ( Table 2), a piece of advice of a salesperson of pharmacy/pharmacist, recalling a previous prescription and a piece of advice of a relative, were the more frequent reasons (38.3%, 25.9%, and 11.7%, respectively).

**Table 2.** Reasons of purchase and responsible individuals of the recommendation to purchase medicines which require medical prescription in Peru without prescription, *Encuesta Nacional de Satisfacción de Usuarios en Salud* (ENSUSALUD) 2016 (N=1892).

	Medicines which require a medical prescription
Questions	n (weighted %)
Why did you purchase medicines without medical prescription? *	
They delay too much to make an appointment to health care in a health center	528 (26.9)
They delay too much to for a health care in a health center	420 (21.5)
Pains are not so serious to go to a physician	431 (20.6)
I do not have money to go to a health center	12 (0.7)
I do not have a health insurance	179 (10.1)
I prefer not to pay for an appointment	148 (8.2)
I think I have enough knowledge to treat myself	114 (6.2)
I took this medicine previously	557 (31.6)
I do not trust in prescriptions of physicians	6 (0.2)
The pharmacy personnel recommends a good treatment	234 (11.0)
Other	38 (2.1)
**Who recommended you the medicines?**	
Nobody, I took them by a personal decision	208 (9.7)
Relative(s)	212 (11.7)
Friend(s)	56 (3.1)
Neighbor(s)	3 (0.1)
Recall of a previous prescription	469 (25.9)
Salesperson of pharmacy/pharmacist	751 (38.3)
Non-medical health personnel (nurse, technician, etc.)	173 (9.0)
Healer/herbalist/bonesetter	3 (0.2)
Other	17 (2.0)

*Question of multiple answer

Bivariate analysis found an association with a value p<0.2 between the purchase of medicines without prescription and all variables of the study ( Table 3). The multivariate analysis showed that being in an age between 25-44 (PRa: 1.11; 95% CI: 1.01-1.22); living in the Highlands region (PRa: 1.39; 95% CI: 1.24-1.57) and Jungle region (PRa: 1.37; 95% CI: 1.23-1.52); and the consumption of purchased products themselves; were associated with a greater prevalence of purchasing medicines without a prescription. Furthermore, having Comprehensive Health Insurance (SIS, for Spanish) (PRa: 0.90; 95% CI: 0.83-0.98) and the pharmacist requesting a prescription (PRa 0.12; 95% CI: 0.10-0.15) were associated with a lower prevalence of purchasing without a prescription for the adjusted model ( Table 3).

**Table 3.** Factors related to the purchase of medicines which required a medical prescription in Peru without prescription, *Encuesta Nacional de Satisfacción de Usuarios en Salud* (ENSUSALUD) 2016 (N=3858).

Characteristic	Crude model *			Adjusted model *		
	PR	(95% CI)	P value	PRa	(95% CI)	P value
**Sex**						
Male	1	-	-	1	-	-
Female	0.89	(0.82-0.96)	0.003	0.96	(0.90-1.03)	0.269
**Age group**						
15–24	1	-	-	1	-	-
25–44	0.95	(0.84-1.06)	0.355	1.11	(1.01-1.22)	0.038
45–59	0.81	(0.70-0.93)	0.003	1.04	(0.93-1.18)	0.476
60 and more	0.83	(0.70-0.98)	0.025	1.05	(0.91-1.22)	0.504
**Level of education**						
Until elementary school	1	-	-	1	-	-
High school	1.00	(0.88-1.15)	0.960	1.01	(0.90-1.14)	0.825
Non-university higher education	1.03	(0.89-1.19)	0.694	0.97	(0.85-1.10)	0.609
University education	1.14	(1.00-1.31)	0.053	0.98	(0.86-1.11)	0.743
**Type of health insurance**						
Without insurance	1	-	-	1	-	-
Comprehensive Health Insurance (SIS)	0.77	(0.68-0.85)	<0.001	0.90	(0.83-0.98)	0.018
EsSalud	0.89	(0.81-0.98)	0.020	0.97	(0.89-1.05)	0.469
Private and other	1.08	(0.87-1.34)	0.511	1.17	(0.98-1.41)	0.082
FF.AA.	0.78	(0.54-1.13)	0.185	1.05	(0.81-1.37)	0.698
**Geographical area of residency**						
Metropolitan Lima	1	-	-	1	-	-
Other areas of Coast region	1.09	(0.95-1.26)	0.214	1.11	(0.98-1.25)	0.096
Highlands region	1.55	(1.35-1.77)	<0.001	1.39	(1.24-1.57)	<0.001
Jungle region	1.53	(1.35-1.73)	<0.001	1.37	(1.23-1.52)	<0.001
**Language**						
Spanish	1	-	-	1	-	-
Other	1.26	(1.01-1.56)	0.038	1.05	(0.86-1.27)	0.638
**Consumption of purchased products**						
For other people	1	-	-	1	-	-
For themselves	1.49	(1.35-1.66)	<0.001	1.21	(1.12-1.31)	<0.001
**Request of prescription by a pharmacist**						
No	1	-	-	1	-	-
Yes	0.11	(0.09-0.14)	<0.001	0.12	(0.10-0.15)	<0.001

*Regression model of Poisson with a log link function, robust variance and weighting of sampling. ^¶^Adjusted by all variables showed in the column and which obtained a value of p<0.2 in crude analysis. PR: prevalence ratio. FF.AA: Health System of Armed Forces and Police.

## Discussion

In this study, a high prevalence of purchasing prescription medicines without medical prescription was found according to the National Authority data. This practice was associated to the age group ranging from 25 to 44 years, inhabitants of the Highlands and Jungle regions, and when the purchase was for self-consumption. Having Comprehensive Health Insurance (SIS) and the pharmacist requesting a prescription would be factors that decrease the prevalence of purchase without prescription. Finally, the advice of the pharmacist, a salesperson of the pharmacy and/or any relative, and the previous use of the medication are cited information sources for the user going to a pharmacy to purchase a medicine without prescription.

Self-medication and the acquisition of medicines without prescription are common worldwide, with a prevalence range varying between 19% and 83% ^3, 16– 18^. The Peruvian natural regions of Highlands and Jungle showed the highest incidences of these practices. Inadequate and non-effective access to medicines has been a common problem in Peru over recent years ^19^, existing in the previously mentioned regions ^7^, which would explain the results found in these regions of the country.

Among the reasons for the purchase of medicines without prescription, the delay to make an appointment or delay in receiving health care at the health center, and previous knowledge of the medicine to be purchased stand out as the most important in our results; this situation was previously reported in national ^20^ and international ^21, 22^ studies. That would reflect the discontent of the users regarding the health service in the Peruvian health centers, where an average of 18 days is expected to make an appointment and 104 minutes to be treated in an outpatient visit after their arrival to the health center for 2015 ^23^.

The practice of purchasing medicines in the surveyed users is mainly focused on self-consumption. It is reported that most of these self-medication practices are developed in the context of self-consumption or for people under someone’s care, like children and elderly people ^4^. A study carried out in eight Latin American countries about responsible self-medication showed that users have a positive attitude towards health care, they recognize that over-the-counter medicines are less dangerous than prescription medicine, they are also interested in reading the labels of the products to be informed about the possible adverse effects ^24^. Thus, with a proper previous education for participants regarding the rational use of medicines, self-medication could produce potential benefits, like the improvement to the access to over-the-counter medicines, greater patient empowerment, and avoidance of unnecessary medical appointments amongst others ^25, 26^.

The control of the pharmacies over the sale of prescription products is important, including antimicrobial medication due to the reported increase in the resistance to these products related to self-medication. This situation occurs both in developed and developing countries ^11, 26, 27^. Regarding a recommendation for the purchase of medicines, the pharmacist or salesperson of the pharmacy was the person that mainly provided this information. This situation has been previously reported in other regions around the world ^3^. Previous conducted studies in the country report the recommendation of medicines other than those in the prescription or a change of prescription, including antibiotics, by the pharmacist or personnel of pharmacies, despite they are not entitled for these practices ^28, 29^. The advice of a relative and the previous consumption of a medicine are other important sources of recommendation for the purchase without prescription. Studies performed in a district of Metropolitan Lima, capital city of Peru, and in Cajamarca, had the same findings ^8, 11^. One of the main problems of this practice is the consumption of medicines that require prescription, such as antibiotics. Antimicrobial resistance has been shown to be associated with inappropriate antibiotic prescription, either for the wrong indication or incorrect duration of treatment ^16, 30, 31^. Allergies to drugs or use of antibiotics to treat viral diseases are other frequent problems ^28, 29^.

From these finding it can be seen that it is crucial to apply measures that guarantee effective access to medicines in health facilities, and to establish procedures that allow verification of proper practices in the purchase of prescription medicines in private pharmacies. The proposals should be an initiative of the national health authority (Ministry of Health) in order to end this phenomenon, taking into consideration the characteristics of people who purchase medicines without prescription.

Among the measures to be included in such a policy are: rationalization of the medicines provision and improvements in the main four stages of the medicines management cycle (selection, purchase, distribution, and use) through actions like: the development of information and control systems; the implementation of proper storage mechanisms and development of clinical practice guidebooks procedures (to promote rational prescription); optimization of the efficiency of investments in supply systems for public provision of medicines; implementation of centralized medication purchases; national and joint international bidding to reduce drug prices; rational prescription, distribution and consumption; among others ^32^.

The national representation of the results and the characterization of each one of the medicines to establish if they require a medical prescription for their purchase are the main strengthens of the study. Also, this is one of the first studies in the country to analyze the relation between socioeconomic factors and the purchase of prescription medicines without medical prescription at national level. However, given that it is a data analysis from secondary sources, there is a possibility that the collected data are not accurate. Because the self-medication item was self-reported causing memory bias, and for drugs for special control, like opioids, causing non-response bias. Another limitation is that it does not allow to establish a causal connection due to the cross-sectional design of the study. Also, ENSUSALUD does not include some morbidity variables in the literature that can affect results ^17^.

Despite that, we consider that our analysis is a good approach to the study of this problem, which represents a greater negative impact in countries with segmented health systems or in developing countries, as in the Peruvian case.

In conclusion, the purchase of prescription medicines without medical prescription is a common practice within the Peruvian health system and is more frequent in regions with a lower supply of medical facilities or access to health services. The development of future programs that allow and/or limit the access to medicines should evaluate the current practices of sale of products without the required prescription in the Peruvian population and the practices of self-medication to strengthen the sale control of these products and improve the access in regions in greater need.

## Data availability

The 2016 *Encuesta Nacional de Satisfacción de Usuarios en Salud* (ENSUSALUD) data are available at from the National Superintendency of Health ( *Superintendencia Nacional de Salud*, SUSALUD) website: http://portal.susalud.gob.pe/blog/base-de-datos-2016/

## Supplementary material

Questionnaires used for this study are available in Spanish from the National Superintendency of Health ( *Superintendencia Nacional de Salud*, SUSALUD) website: http://portal.susalud.gob.pe/wp-content/uploads/archivo/encuesta-sat-nac/2016/Cuestionario-Usuarios-en-boticas-y-farmacias.pdf
