# Supplementary material for: Purchase of medications without prescription in Peru: a cross-sectional population-based study
Source: F1000Res. 2019 Feb 22;7:1392. Originally published 2018 Sep 3. [Version 2] doi: 10.12688/f1000research.15886.2 (PMC6364375; doi:10.12688/f1000research.15886.2)
Supplement: Peer review history

# Peer review: Reviewer response for version 2

**DOI:** 10.5256/f1000research.19921.r44809

**Published:** 2020-01-27

**Authors:** Marcus Tolentino Silva

**Affiliations:** 1 Post-Graduate Program of Pharmaceutical Science, University of Sorocaba, Sorocaba, Brazil

I have no further comments to make.

I confirm that I have read this submission and believe that I have an appropriate level of expertise to confirm that it is of an acceptable scientific standard.

# Peer review: Reviewer response for version 2

**DOI:** 10.5256/f1000research.19921.r44810

**Published:** 2019-03-08

**Authors:** Sandipan Bhattacharjee

**Affiliations:** 1 Department of Pharmacy Practice and Science, College of Pharmacy, University of Arizona, Tucson, AZ, USA

The revised version looks much better and I have no further comments. This manuscript can now be accepted for indexing.

I confirm that I have read this submission and believe that I have an appropriate level of expertise to confirm that it is of an acceptable scientific standard.

# Peer review: Reviewer response for version 2

**DOI:** 10.5256/f1000research.19921.r44808

**Published:** 2019-02-22

**Authors:** Fernando M. Runzer-Colmenares

**Affiliations:** 1 Universidad Cientifica del Sur, Lima, Peru

I consider this version as approved.

I confirm that I have read this submission and believe that I have an appropriate level of expertise to confirm that it is of an acceptable scientific standard.

# Peer review: Reviewer response for version 1

**DOI:** 10.5256/f1000research.17344.r43615

**Published:** 2019-02-04

**Authors:** Fernando M. Runzer-Colmenares

**Affiliations:** 1 Universidad Cientifica del Sur, Lima, Peru

Interesting article, finely written. Introduction reports adequately about antecedents and justification of research. Methods reports designs, variables, and statistical analysis. Results and analysis are performed adequately in my opinion and discussion is enriched with comparison with recent publications about the research question, that's why I consider this paper as suitable for publication. The authors may detail the criteria used to include variables in the regression model.

I confirm that I have read this submission and believe that I have an appropriate level of expertise to confirm that it is of an acceptable scientific standard.

# Peer review: Reviewer response for version 1

**DOI:** 10.5256/f1000research.17344.r38798

**Published:** 2019-01-23

**Authors:** Marcus Tolentino Silva

**Affiliations:** 1 Post-Graduate Program of Pharmaceutical Science, University of Sorocaba, Sorocaba, Brazil

This is a nationwide based study of Peru that measured self-medication by adults (>15 years old) in 2016. The authors showed that poor access to health services increases self-medication. I would like to add three suggestions. First, investigate colinearity between independent variables to avoid type I errors. Second, discuss the measurement bias: the self-medication was self-reported and may be influenced by memory bias (last 12 months) or non-response bias (for drugs for special control, like opioids, etc.). Third, add reference from Latin America ^1^.

I confirm that I have read this submission and believe that I have an appropriate level of expertise to confirm that it is of an acceptable scientific standard.

## References

[1] : Prevalence of self-medication in the adult population of Brazil: a systematic review. *Revista de Saúde Pública*.2015;49(0) : 10.1590/S0034-8910.2015049005709 10.1590/S0034-8910.2015049005709 26083944PMC4544343

# Peer review: Reviewer response for version 1

**DOI:** 10.5256/f1000research.17344.r41709

**Published:** 2018-12-27

**Authors:** Sandipan Bhattacharjee

**Affiliations:** 1 Department of Pharmacy Practice and Science, College of Pharmacy, University of Arizona, Tucson, AZ, USA

The study titled "Purchase of medications without prescription in Peru: a cross-sectional population-based study" is an interesting and relevant study from the perspective of Peru. The authors have done a good job. However, some of the following issues should be addressed prior to indexing.

1. In the abstract, the author mention age range of 24-45, but in the study they have used 25-44 years. This should be addressed.
2. I would suggest suing the Jungle region term consistently in the abstract as well (instead of Amazon).
3. In the second paragraph of the Introduction, the authors mention "several studies" but only provide two studies as reference (1, 4). If it is only two studies, then it should be called a few or handful studies. Also, where were these studies conducted and how are they relevant in the context of Peru?
4. In the third paragraph of the Introduction section, some important statistics are provided. But it might be more appropriate to mention when these changes happened etc.
5. The weighted percentage column of Table 1 is confusing. Are they column percentages? If so, then why are they not adding up to 100%. The authors need to clarify this issue.
6. In Table 2, there is a mention of "Other" reason to purchase medicines without medical prescription. I am wondering if the respondents were allowed to provide comments on them? If yes, then some interesting comments should be provided either in this Table or in the text somewhere in the Results section.
7. And finally, although this manuscript is rather well written, but in some places English editing is required for clarification (e.g. - second sentence of the Methods section of Abstract is not very clear to me).

I confirm that I have read this submission and believe that I have an appropriate level of expertise to confirm that it is of an acceptable scientific standard, however I have significant reservations, as outlined above.
